# Evidence‐based selection of reference genes for RT‐qPCR assays in periodontal research

**DOI:** 10.1002/cre2.525

**Published:** 2022-02-01

**Authors:** Daniel Diehl, Anton Friedmann, Hagen S. Bachmann

**Affiliations:** ^1^ Center for Biomedical Education and Research (ZBAF), Institute of Pharmacology and Toxicology, Faculty of Health Witten/Herdecke University Witten Germany; ^2^ Department of Periodontology, School of Dentistry, Faculty of Health Witten/Herdecke University Witten Germany

**Keywords:** genetic research, methods, periodontics, quantitative real‐time PCR

## Abstract

**Objective:**

To underline the necessity of adequate reference genes for real‐time quantitative polymerase chain reaction (RT‐qPCR) and evaluate a novel tool for condition‐specific reference gene selection.

**Background:**

RT‐qPCR is a commonly used experimental technique that allows for highly sensitive analysis of gene transcription. Moreover, the use of internal reference genes as a means for relative quantification has rendered RT‐qPCR a straightforward method for a variety of sciences, including dentistry. However, the expressional stability of internal reference genes must be evaluated for every assay in order to account for possible quantification bias.

**Materials and Methods:**

Herein, we used the software tool RefGenes to identify putatively stable reference genes with the help of microarray datasets and evaluated them. Additionally, we propose an evidence‐based workflow for adequate normalization of thusly identified genes. Human gingival fibroblasts (HGF‐hTert), human acute leukemia‐derived monocytes (THP‐1), and telomerase immortalized gingival keratinocytes (TIGKs) were subjected to set‐ups simulating various glycemic conditions and lipopolysaccharide challenges. Five common housekeeping genes (HKGs) and five genes from RefGenes were selected as targets and RT‐qPCR was performed subsequently. Then, normalization algorithms Bestkeeper, Normfinder, and geNorm were used for further analysis of the putative reference gene stability.

**Results:**

RefGenes‐derived targets exhibited the highest stability values in THP‐1 and TIGK cell lines. Moreover, unacceptable standard variations were observed for some common HKG like β‐actin. However, common HKG exhibited good stability values in HGF‐hTert cells.

**Conclusion:**

The results indicate that microarray‐based preselection of putative reference genes is a valuable refinement for RT‐qPCR studies. Accordingly, the present study proposes a straightforward workflow for evidence‐based preselection and validation of internal reference genes.

## 1 INTRODUCTION

Periodontitis is a chronic multifactorial inflammatory disease associated with dysbiotic plaque biofilms and characterized by progressive destruction of the tooth‐supporting apparatus (Van Dyke & Dave, 2005). In the postgenome era, molecular periodontal research has become capable of elucidating the individual susceptibility and the pathogenesis of this disease in terms of the host‐derived immune reaction. Hence, biochemical techniques measuring gene transcription have been increasingly utilized in periodontal research, the most popular being real‐time quantitative polymerase chain reaction (RT‐qPCR). RT‐qPCR is a routine method for the quantification of messenger RNA (mRNA) in biological samples, which constitutes a high sensitivity and a large dynamic range. This high sensitivity makes it highly beneficial for periodontal research, as the yield of biological samples is often small and immunologic target genes are typically not expressed in abundance.

Nonetheless, a variety of issues are associated with the establishment of a reliable RT‐qPCR assay, including the variability of RNA quantity between samples and differing reverse transcription or PCR efficiencies (Bustin & Nolan, 2004). Therefore, normalization is crucial to obtain valid relative gene expression results. This is commonly accomplished by using one or more internal reference genes and the 2^−ΔΔCq^ method (Schmittgen & Livak, 2008) for the calculation of a relative fold change. The internal reference and the gene of interest are used in the same assay. Thereby, all the aforementioned issues are accounted for (Huggett et al., 2005). As a matter of consequence, the internal reference genes should, theoretically, exhibit stable expression patterns across all tissues and experimental conditions to obtain accurate fold changes (Vandesompele et al., 2002).

Housekeeping gene (HKG) is a term that refers to ubiquitously expressed genes that are required for the maintenance of basic cellular functions. Commonly utilized HKGs are glyceraldehyde‐3‐phosphate dehydrogenase (*GAPDH*), β‐actin (*ACTB*), hypoxanthine‐guanine phosphoribosyltransferase (*HPRT*), β‐2‐microglobulin (*B2M*), and succinate dehydrogenase complex, subunit A (*SDHA*) (de Jonge et al., 2007; Huggett et al., 2005; Klenke et al., 2016; Schmittgen & Zakrajsek, 2000). Despite the underlying theory, the use of HKG as an internal reference has been shown to be biased and error‐prone over the course of the last two decades, mainly due to substantial expression variability in different experimental conditions (Glare et al., 2002; Schmittgen & Zakrajsek, 2000; Selvey et al., 2001). According to the Minimum Information for publication of Quantitative Real‐Time PCR experiments (MIQE) guideline, published by Bustin et al. (2009), stable expression of reference genes across experimental conditions must be validated and reported before a new investigation. In context to this, various papers disclose adequate reference genes for specific experiments or cells and introduce free‐to‐use normalizing algorithms like NormFinder, Bestkeeper, and geNorm (Andersen et al., 2004; Pfaffl et al., 2004; Vandesompele et al., 2009). However, the choice of genes to be validated, therein, remains somewhat arbitrary to this day. To address this issue, Hruz et al. (2011) introduced the RefGenes tool, an application built into Genevestigator (Hruz et al., 2008) software, which provides a genome‐wide selection of the most stable reference genes on the basis of microarray data according to the studied organism, experimental condition, and cell line. In a series of experiments, they were able to demonstrate that condition‐specific selection of reference genes outperformed arbitrary HKGs in terms of expression stability.

Thus, the aim of the following study was to (1) underline the issues associated with unvalidated use of reference genes, (2) evaluate the expression stability of widespread HKG‐ and RefGenes‐proposed genes by means of common data normalization algorithms, and to (3) introduce a straightforward approach to evidence‐based reference gene selection in future periodontal research.

## 2 MATERIALS AND METHODS

### 2.1 Selection of reference genes and primer design

To address the aforementioned hypothesis, we compared a set of five commonly used HKGs to five putatively stable genes recommended by the RefGenes algorithm (Nebion AG, Zurich, Switzerland). Reviewing the literature, we identified *GAPDH, ACTB, B2M, HPRT1*, and *SDHA* as widely used, common HKG (Cummings et al., 2014; Kozera & Rapacz, 2013; Tricarico et al., 2002)

In order to account for the experimental conditions and the utilized cell lines, we used the RefGenes tool to identify potential reference genes across 166 mRNA‐sequencing arrays (Figure S1) mapping closely related cell types in six studies overall (Elbediwy et al., 2016; Fleischer et al., 2018; Ghandi et al., 2019; Letourneau et al., 2014; Serezani et al., 2017; Torán et al., 2017). Primers for selected target genes were designed with NCBI PrimerBlast (Ye et al., 2012) on the basis of mRNA data derived from the NCBI Reference Sequence database (RefSeq, National Center for Biotechnology Information, Bethesda MD, USA). All primers were compelled to contain an exon–exon junction and amplicon lengths of 70–200 nucleotides (Table 1). Oligonucleotides were ordered from Eurofins MWG Operon LLC (Eurofins Scientific SE, Luxembourg City, Luxembourg). The reference genes proposed by RefGenes that met with our mentioned stipulation were *PSMB4, EEF1B2, RPL10A, PPIA*, and *ARPC3* (Table 1).

**Table 1:** Gene names, sequence disclosure, and amplicon information for RT‐qPCR primers

Gene	Accession number	Gene name	5′→3′ Forward primer (*T*_m_/%GC)	5′→3′ Reverse primer (*T*_m_/%GC)	Amplicon length (bp)	Amplification efficiency (%)
*ACTB*	NM_001101.5	Actin beta	GCACAGAGC	TATCATCATCCA	70	92.24
			CTCGCCTTT	TGGTGAGCTGG		
			(60.36°C/61.11%)	(59.99°C/47.83%)		
*ARPC3*	NM_001278556.2	Actin‐related protein 2/3	CCTGGTTTTCC	AATAGGCTCTCA	73	104.26
			ACTTAACGCA	TCACTTCATCTT		
			(59.05°C/47.62%)	(57.50°C/37.50%)		
*B2M*	NM_004048.4	β‐2‐microglobulin	TGAGTATGC	GCTTACATGTCT	75	109.52
			CTGCCGTGTG	CGATCCCACT		
			(60.08°C/57.89%)	(59.90°C/50.00%)		
*EEF1B2*	NM_021121.4	Eukaryotic elongation factor 1 beta 2	TTCGGAGAC	GTGATGGCAC	88	112.49
			CTGAAAAGCCC	ATACCCCTCG		
			(59.96°C/55.00%)	(60.53°C/60.00%)		
*GAPDH*	NM_001357943.2	Glyceraldehyde‐3‐phosphate dehydrogenase	AAGGTGAAG	TTCCCGTTCTCA	137	94.47
			GTCGGAGTCAAC	GCCATGTAGT		
			(59.93°C/52.38%)	(61.41°C/50.00%)		
*HPRT1*	NM_000194.3	Hypoxanthine phosphoribosyltransferase 1	CCCTGGCGTC	CACCCTTTCCA	91	100.80
			GTGATTAGTG	AATCCTCAGC		
			(60.81°C/60.00%)	(59.18°C/52.38%)		
*PSMB4*	NM_002796.3	Proteasome 20S subunit beta 4	TCGGCCAGAT	CAGCATAGCC	150	106.33
			GGTGATTGAT	TCCGATGACC		
			(59.16°C/50.00%)	(60.32°C/60.00%)		
*PPIA*	NM_001300981.2	Peptidylprolyl isomerase A	GCTGTTTACCC	CCTTGTCTGCA	70	88.41
			CTGATCGTG	AACAGAAGGCA		
			(58.63°C/55.00%)	(61.59°C/50.00%)		
*RPL10A*	NM_007104.5	Ribosomal protein L10a	TGAGCAGCAA	GTGGACTTA	172	91.71
			AGTCTCTCGC	AGCCTGACGGT		
			(60.67°C/55.00%)	(59.68°C/55.00%)		
*SDHA*	NM_001330758.2	Succinate dehydrogenase complex flavoprotein subunit A	GCATTTGGCCT	TTGATTCCTCC	95	94.47
			TTCTGAGGC	CTGTGCTGC		
			(60.11°C/55.00%)	(60.32°C/55.00%)		

Abbreviations: %GC, GC content; RT‐qPCR, real‐time quantitative polymerase chain reaction; *T*_m_, melting temperature.

### 2.2 Cell culture

Three cell lines were used for in vitro experiments. Human monocytes of the acute leukaemia (THP‐1) cell line (CLS, Eppelheim, Germany) were cultured in RPMI‐1640 medium (PAN Biotech, Aidenbach, Germany), supplemented with 10% fetal bovine serum (PAN‐Biotech), 100 U/ml penicillin, and 100 µg streptomycin. Approximately 0.7 × 10^6^ THP‐1 monocytes were seeded into T‐25 cell culture flasks. A concentration of 50 nM phorbol‐12‐myristate‐13‐acetate (PMA; Biomol, Hamburg, Germany) was added to the medium for differentiation into macrophage‐like cells according to Bylski et al. (2009) and Jablonski et al. (2015). Cells were cocultured with PMA until they exhibited visual adherence to the culture flask.

After differentiation, the cells were washed once in Dulbecco's phosphate‐buffered saline (DPBS; PAN‐Biotech) and seeded into 12‐well culture plates in PMA‐free medium at a density of 1 × 10^5^ cells for further experiments. Human telomerase reverse‐transcriptase immortalized human gingival fibroblasts (HGF‐hTert; Applied Biological Materials Inc., Richmond, BC, Canada) were cultured in Dulbecco's modified Eagle's medium (PAN‐Biotech) with the supplements mentioned above. At 85% confluence, the cells were washed in DPBS, suspended in a fresh culture medium, and seeded into 12‐well culture plates for consecutive experiments. Telomerase immortalized gingival keratinocyte cell line (TIGK‐hTert; ATCC CRL‐3397, Manassas, VA, USA) was cultured in Dermal Cell Basal Medium (ATCC) and supplemented with Keratinocyte Growth Kit (ATCC PCS‐200‐040). Keratinocytes were prepared for consecutive experiments in the aforementioned manner.

All cell lines were incubated in a humidified 5% CO_2_ environment at 37°C. Muse Count & Viability assay (Merck Millipore, Burlington, MA, USA) determined cell count and viability before seeding into 12‐well plates.

### 2.3 In vitro experiments

**Figure 1:**
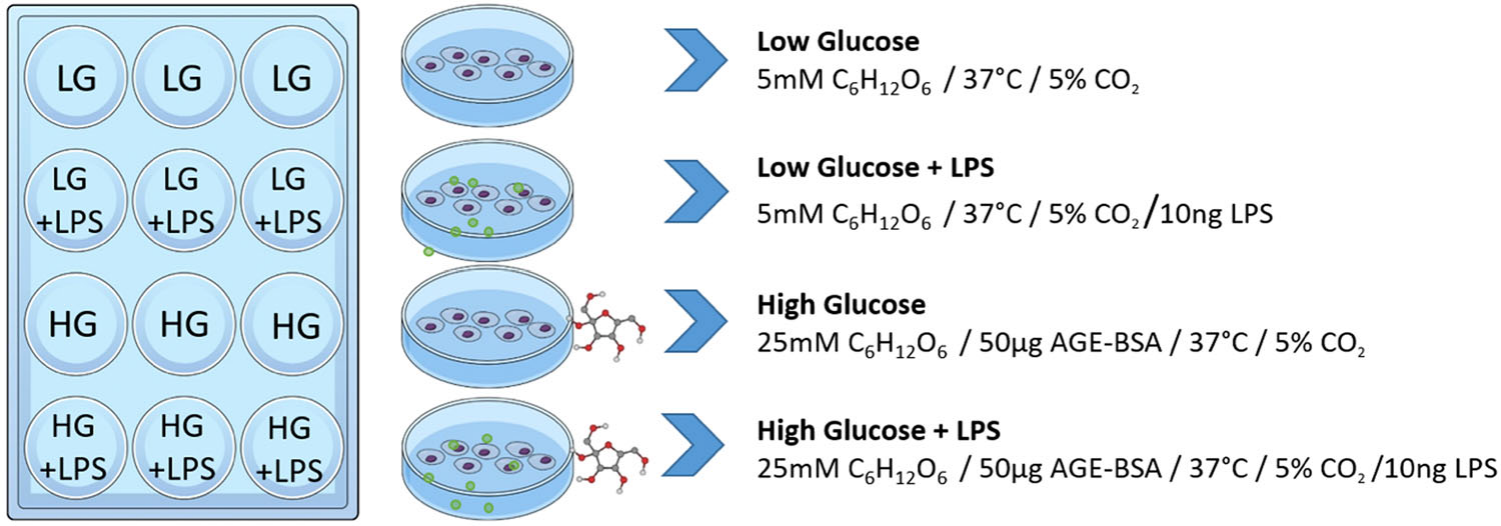
Cell culture setup for all experimental conditions. Each condition was replicated three times for each cell line (*n* = 3). AGE‐BSA, advanced glycation endproducts‐bovine serum albumin; HG, high glucose; LG, low glucose; LPS, lipopolysaccharide

To simulate different metabolic environments, each cell line was cultured in either hyperglycemic or normoglycemic conditions. The hyperglycemic environment was simulated by adding 4500 µg/ml glucose instead of 1000 µg/ml to the medium. Furthermore, advanced glycation endproducts were prepared from bovine serum albumin according to Waanders et al. (2007) and added to the high glucose culture medium at a final concentration of 50 µg/ml. Each culture plate consisted of six low glucose wells and six high glucose wells (Figure 1). Upon confluence, lipopolysaccharide (LPS) (Sigma‐Aldrich, Taufkirchen, Germany) was administered to three of six wells in each metabolic condition at a concentration of 100 ng/ml. After 24 h of incubation, the cells were washed in DPBS and collected for RNA preparation.

### 2.4 RNA preparation and purity assessment

Total RNA isolation was performed with the RNEasy Mini Kit (Qiagen, Hilden, Germany) according to the manufacturer's instructions. Because all administered oligonucleotide primers were designed spanning exon–exon junctions, no DNase treatment was carried out but a no‐reverse‐transcriptase control (NRT) was used in all experiments.

RNA was eluted in 30 µl of nuclease‐free water. Subsequently, optical density (OD) was measured photometrically at 260 and 280 nm using a Tecan Infinite200 PRO plate reader (Tecan Trading AG, Männedorf, Switzerland). An OD_260 nm/280 nm_ of 1.9–2.1 was considered protein‐free RNA (Table S1). All RNA samples were stored at −80°C until further use.

### 2.5 Synthesis of complementary DNA (cDNA)

For cDNA synthesis, we used the PrimeScript RT Master Mix (Takara Bio, Kusatsu, Japan) according to the manufacturer's instructions. For each cDNA synthesis, a quantity of 1 µg RNA was added to a volume of 4 µl of 5× PrimeScript RT Master Mix (Takara Bio) and stocked up to 20 µl with RNAse‐ree ddH_2_O (Takara Bio). The Thermocycler T100 (Bio‐Rad Laboratories, Hercules, CA, USA) was set to a reverse transcription step of 15 min at 37°C, a 5‐s inactivation step at 85°C, and holding temperature at 4°C, according to the manufacturer's protocol. The cDNA samples were immediately stored at −20°C.

### 2.6 Assay and amplification validation

**Figure 2:**
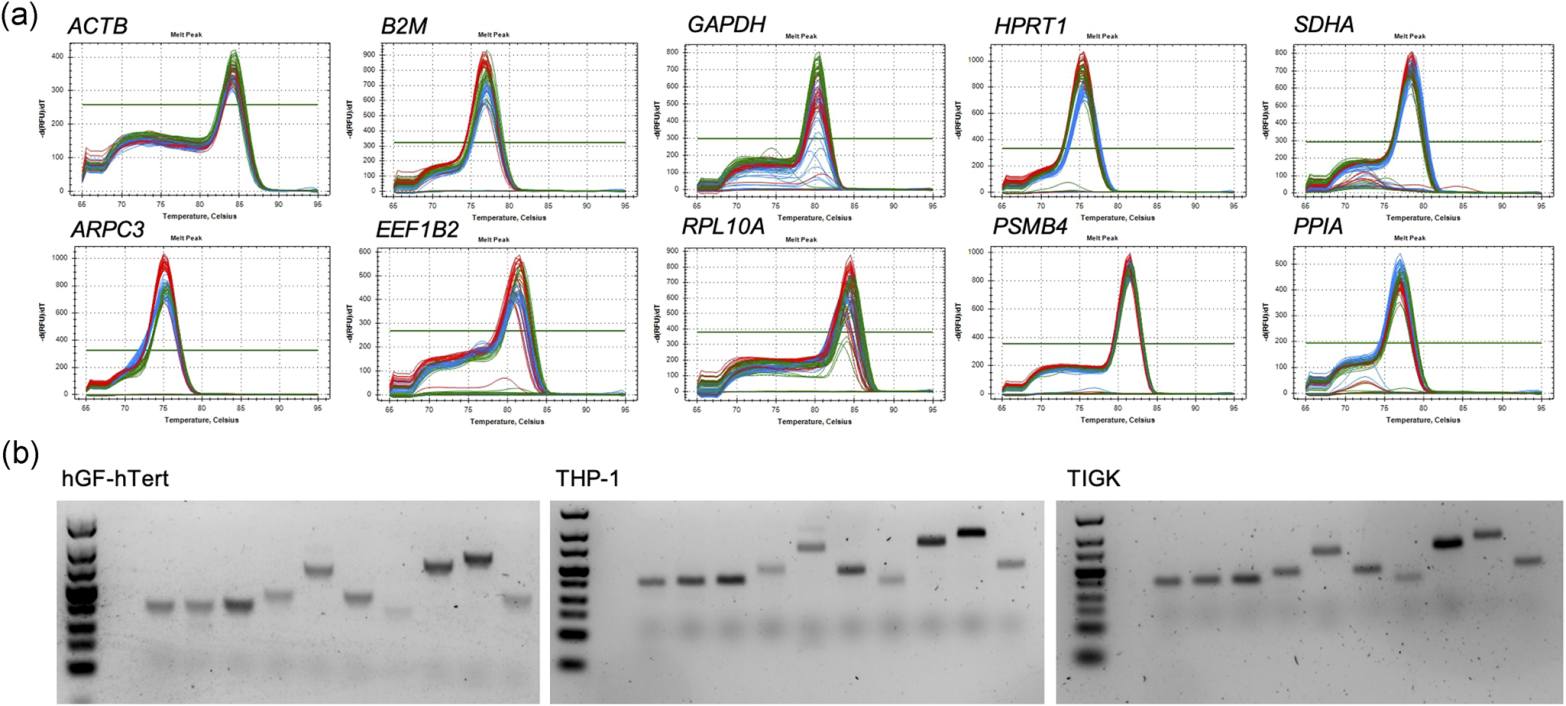
Amplicon specificity for candidate reference genes determined by (a) melting curve analysis and (b) agarose gel electrophoresis. Gel images are cropped. Full‐length gel images are available in the Supporting information Material. ACTB, β‐actin; B2M, β‐2‐microglobulin; GAPDH, glyceraldehyde‐3‐phosphate dehydrogenase; HPRT, hypoxanthine‐guanine phosphoribosyltransferase; SDHA, succinate dehydrogenase complex, subunit A; HGF‐hTert, human telomerase reverse‐transcriptase immortalized human gingival fibroblasts; THP‐1, human acute leukemia‐derived monocytes; TIGK, telomerase immortalized gingival keratinocytes

Primer efficiency *E*_p_ was determined by means of a 6× log_10_ serial dilution of a pooled standard cDNA solution derived from the three untreated cell lines. Each candidate reference gene was amplified in three technical replicates at their respective annealing temperature *T*_a_ (Tables S2–11) and a standard curve was created by means of linear regression analysis in PRISM 8 software (GraphPad Software, San Diego, CA, USA). The coefficient of determination (*r* ^2^) was calculated from the respective linear regression and primer efficiency (*E*_p_) was derived from the slope: *E*_p_ = 10^−1/slope^ − 1. Acceptable *E*_p_ and *r* ^2^ thresholds within the linear dynamic range (LDR) were predefined at 90%–110% and *r* ^2^ > .97, respectively. The limit of detection was defined as the dilution factor above the serial dilution at which the regression stops being linear (Figures S3–11). The assay specificity for each gene of interest was evaluated by melting curve analysis, with a singular peak signifying primer specificity (Figure 2). Bias from contaminating genomic DNA or primer dimers was assessed with a no‐template control for each sample and an NRT containing RNA instead of cDNA.

### 2.7 RT‐qPCR

For qPCR amplification, a CFX96 Touch Real‐Time PCR cycler (Bio‐Rad) was used with 96‐well Hard Shell PCR plates (Bio‐Rad). Twenty microliters of Master Mix, consisting of 2× iTaq Universal SYBR® Green Supermix (10 µl, Bio‐Rad), the respective cDNA solution (1 µl), the respective primer pair (10 pmol/0.3 µl per primer), and nuclease‐free H_2_O (8.4 µl; Fresenius Kabi, Bad Homburg, Germany) was administered per well. The plates were covered with Nunc sealing tape (Nalge Nunc International, Rochester, NY, USA). Amplification of three biological replicates was carried out in technical duplicates for each gene of interest and every setup. A C1000 Touch Thermal Cycler in conjunction with the CFX96 Real‐Time System (Bio‐Rad) was used. This resulted in 36 analyzed PCR reactions per candidate reference gene (3 biological replicates × 2 technical replicates × 4 experimental conditions). The amplification protocol (40 cycles, initial denaturation at 95°C/3 min, denaturation at 95°C/10 s, annealing at the respective *T*_a_/30 s, extension at 70°C/10 s) was followed by a consecutive melting curve analysis (65–95°C in 0.5°C increment/5 s each).

### 2.8 Statistical analysis

The quantification cycle (Cq) values and melt curves were calculated within the CFXMaestro software (Bio‐Rad). Arithmetic means of each Cq triplet were used in further analysis. Three different algorithms were used for the determination of reference gene stability: geNorm, Bestkeeper, and Normfinder (Andersen et al., 2004; Pfaffl et al., 2004; Vandesompele et al., 2009). For geNorm calculations, the qBase+ software package was used (Biogazelle, Zwijnaarde, Belgium) and the free‐to‐use excel spreadsheet applications were used for the other two algorithms. While the Bestkeeper application performs calculations upon raw Cq values, the Normfinder and geNorm algorithms require date transformed into linear scale expression quantities. This was accomplished by the primer efficiency (*E*_p_) cleared 2^−ΔCq^ method after calculating fold changes for the lowest Cq (Cq_min_) of each gene ($E_{p}-({\mathrm{Cq}}_{\min}-{\mathrm{Cq}}_{\mathrm{sample}})$). Calculations were performed according to protocol. After that, rank sums were allocated to each gene, in order to account for different underlying mathematical principles, which may result in dissimilar ranking orders. At last, the three most stable reference genes were subjected to the Bestkeeper excel sheet to calculate a weighted Bestkeeper index and to check for each gene's correlation to it via pairwise correlation analysis (Pfaffl et al., 2004).

**Figure 3:**
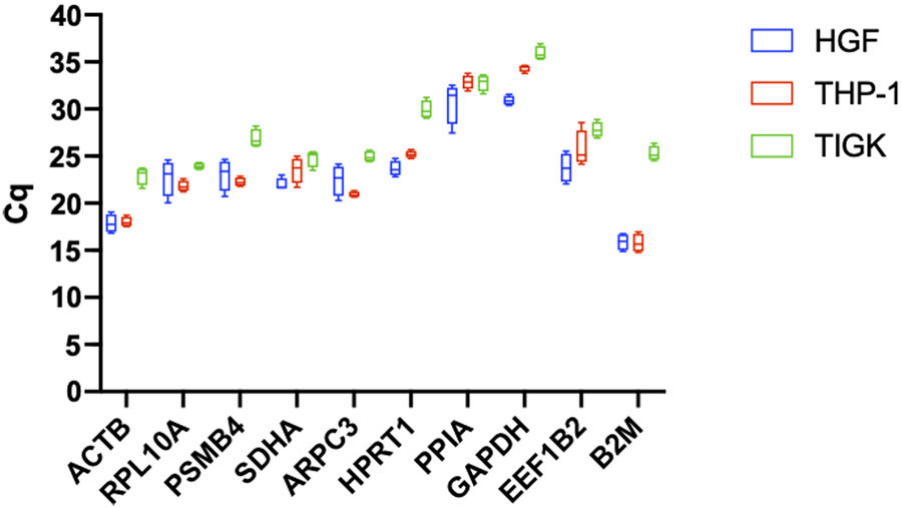
Mean quantification cycles (Cq) from tested reference genes across all experimental conditions (*n* = 12). Boxplots show median, interquartile range (box), and minimum and maximum values (whiskers). HGF‐hTert, human telomerase reverse‐transcriptase immortalized human gingival fibroblasts; THP‐1, human acute leukemia‐derived monocytes; TIGK, telomerase immortalized gingival keratinocytes

In order to evaluate the expectable impact of different reference genes on relative gene expression data, the least stable gene according to the calculated rank sums (Figure 3) served as a hypothetical target. Accordingly, *EEF1B2* served as the target in the fibroblast cell line, *ACTB* in the keratinocyte cell line, and *B2M* in the monocyte cell line. Next, expression changes were calculated by means of the efficiency‐corrected 2^−(ΔΔCq)^ method (Schmittgen & Livak, 2008) using the CFXMaestro GeneStudy tool (Bio‐Rad). Calculations were performed with either a common HKG or the experimentally determined reference gene index serving as the internal control. Furthermore, the low glucose condition (LG) served as the control group, while the other aforementioned conditions (Figure 1) were used as hypothetical test groups. The expression data are presented as 2^−(ΔΔCq)^ fold changes with standard error of the mean.

## 3 RESULTS

### 3.1 RNA quantities, primer specificity, amplification efficiency, and quantification

All RNA quantities and OD_260/280_ ratios are provided in Table S1). The amplicon specificity was evaluated by melting curve analysis (Figure 2a), which indicated a single peak for every target. Furthermore, specific PCR products were confirmed by agarose gel electrophoresis (Figure 2b), yielding one single band at the expected size for each gene in all experimental conditions. The RT‐qPCR efficiencies ranged from 91.71% (*RPL10A*) to 109.57% (*GAPDH*) (Table 1). The linear regressions for serial dilutions of each gene are provided in the Supporting information. The mean Cq values, which are inversely proportional to the amount of template cDNA in the original sample, ranged from 14.89 (*B2M* in HGF cells) to 36.91 (*GAPDH* in TIGK cells). The mean Cq ranges across all experimental conditions for all genes investigated are depicted in Figure 3.

### 3.2 Evaluation of expression stability

**Figure 4:**
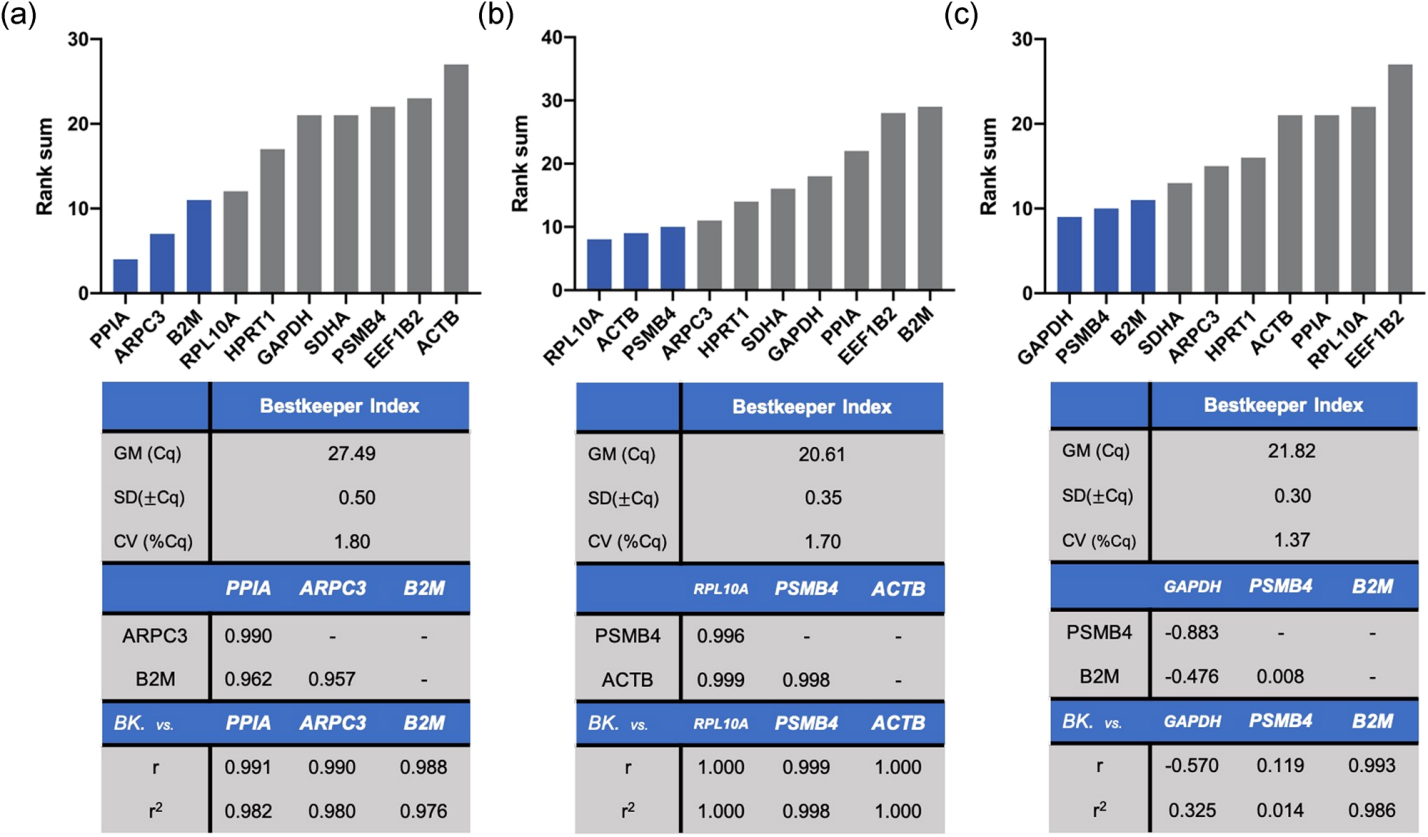
Overall results for the proposed normalization approach. (a) Telomerase immortalized gingival keratinocytes. (b) Macrophage‐like human acute leukemia‐derived monocyte (THP‐1) cells. (c) Human gingival fibroblasts. Graphs show rank sums of three different normalization algorithms Normfinder, Bestkeeper, and geNorm. The associated table presents keystone data for Bestkeeper calculations. BK, Bestkeeper; Cq, mean quantification cycles; CV, coefficient of variation; GM, geometric mean; *r*, coefficient of correlation; *r* ^2^, coefficient of determination; SD, standard deviation (Pfaffl et al., 2004)

**Figure 5:**
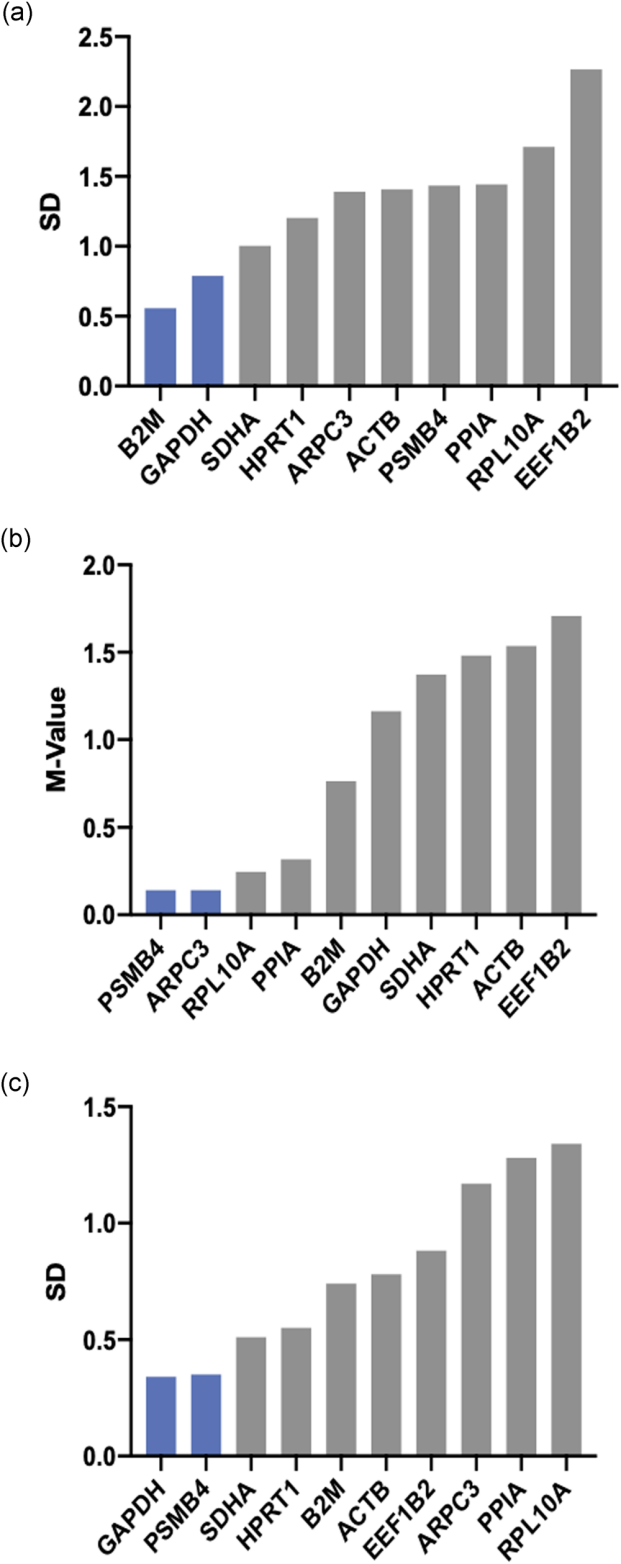
Ranking orders for expression stability by means of different normalization algorithms in human telomerase reverse‐transcriptase immortalized human gingival fibroblasts (HGF‐hTert) cells. Normfinder (a) and Bestkeeper (c) calculate standard deviations (SDs) across experimental conditions. GeNorm (b) calculates the *M* value from pairwise mean quantification cycle (Cq) variation

**Figure 6:**
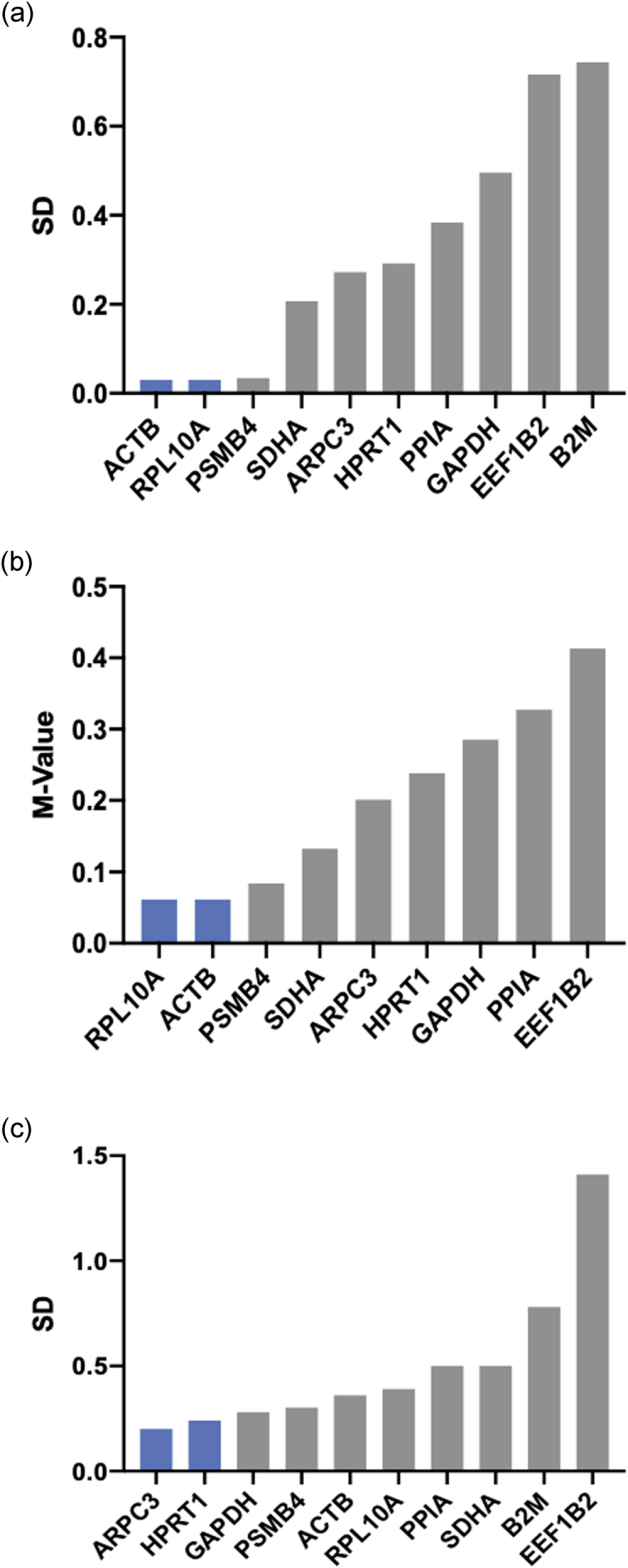
Ranking orders for expression stability by means of different normalization algorithms in human acute leukemia‐derived monocyte (THP‐1) cells. Normfinder (a) and Bestkeeper (c) calculate standard deviations (SDs) across experimental conditions. GeNorm (b) calculates the *M* value from pairwise mean quantification cycle (Cq) variation

**Figure 7:**
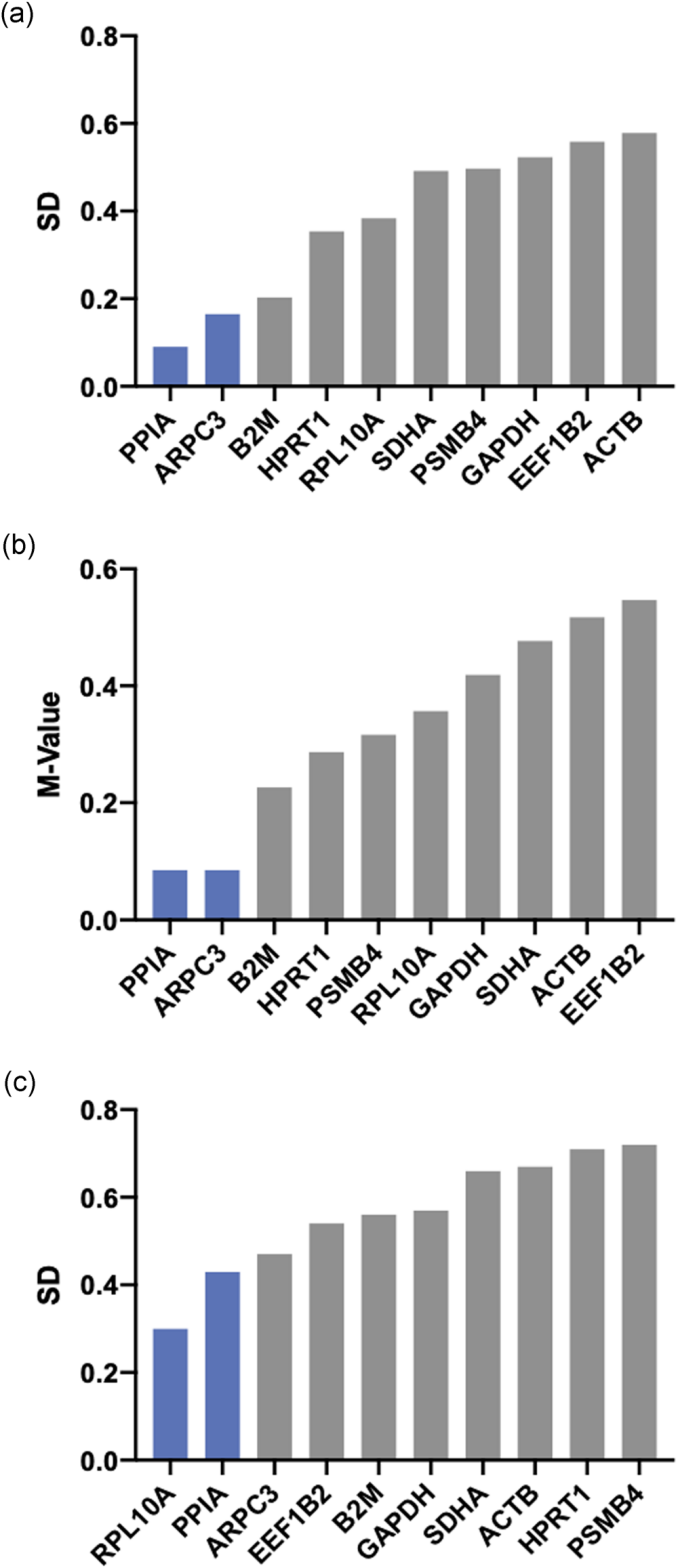
Ranking orders for expression stability by means of different normalization algorithms in telomerase immortalized gingival keratinocyte (TIGK) cells. Normfinder (a) and Bestkeeper (c) calculate standard deviations (SD) across experimental conditions. GeNorm (b) calculates the *M* value from pairwise mean quantification cycle (Cq) variation

Figure 4 displays the overall order of expression stability as rank sums. Across all experimental conditions, we identified *PPIA*, ARPC, and *B2M* to be the most stably expressed genes in the keratinocyte cell line TIGK. Moreover, *ACTB*, which is utilized for normalization quite frequently, was ranked the least stable gene to be used for this setup. In the subset of macrophage‐like THP‐1 cells, the recommended reference genes were *RPL10A, ACTB*, and *PSMB4*. On the other hand, it was revealed that *GAPDH* and *PSMB4* along with *B2M* exhibited the highest expression stability in terms of the gingival fibroblast cell line (Figure 5). The stability ranking order for separate algorithms is provided in Figures 5, 6, 7.

### 3.3 Proposed approach for adequate reference gene selection

**Figure 8:**
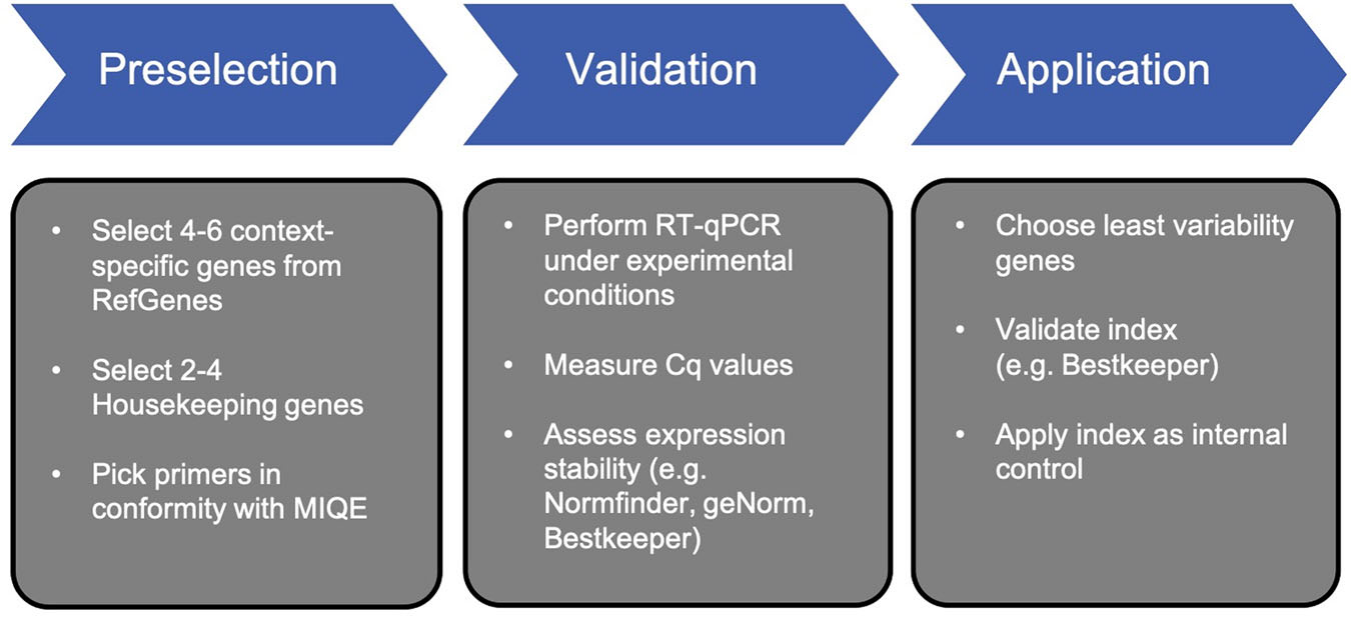
Suggestion of a straightforward and evidence‐based workflow for finding and validating adequate reference genes for RT‐qPCR experiments. Cq, mean quantification cycle; MIQE, Minimum Information for publication of Quantitative Real‐Time PCR experiments; RT‐qPCR, real‐time quantitative polymerase chain reaction

The suggested workflow (Figure 8) summarizes our efforts to find a reliable way of identifying adequate reference genes, which is applicable to a variety of experimental conditions. The first step of this approach is to preselect a set of putative reference genes from a specific perturbation, tissue or cell line in RefGenes (Hruz et al., 2011). It is to be noted that common HKG may be adequate under certain conditions, as we were able to elucidate in this study. For that reason, adding HKGs to the context‐specific recommendations posed by Genevestigator software may be beneficial.

Next, RT‐qPCR is performed with all samples under intended experimental conditions and obtained Cq values are analyzed by normalization algorithms. Using at least three different algorithms is recommended to avoid bias, as the underlying calculations may influence the ranking order (Jacob et al., 2013). Nonetheless, adequate reference genes should not only exhibit low cross‐experimental variation but also an adequate correlation with each other in terms of expression patterns (Pfaffl et al., 2004). To account for this, we advise to reevaluate correlation among the least‐variability reference genes by means of the Bestkeeper algorithm. With respect to this study, the resulting indexes displayed small standard deviations, ranging from 0.30 to 0.50 Cq, while correlating well with each other in TIGK and THP‐1 cells (.976 < *r* ^2^ < 1.000; Figure 4).

### 3.4 Impact of reference genes on expression data

**Figure 9:**
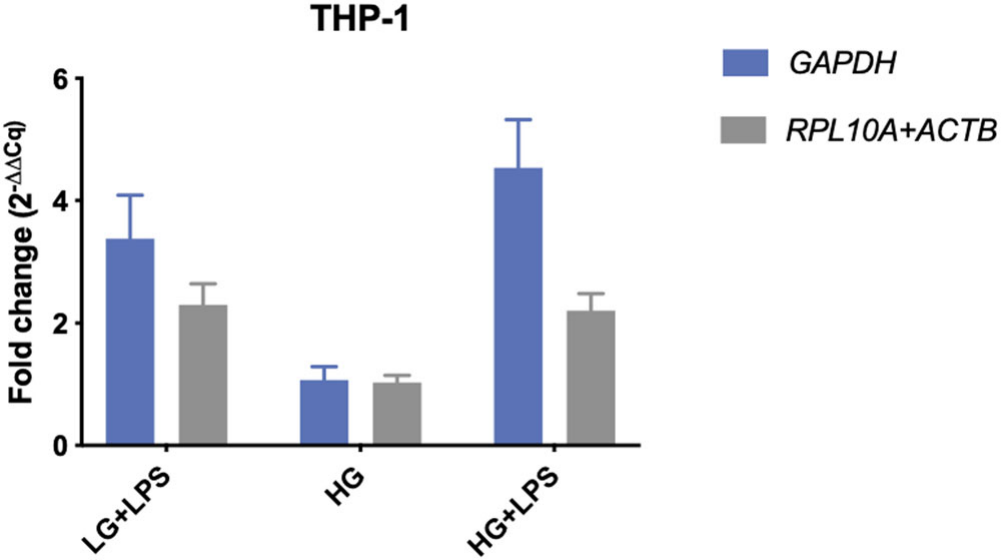
Expression fold changes (2^(−ΔΔCq)^) of *B2M* between three experimental conditions and the low glucose (LG) group. Calculations were performed with a common housekeeping gene and a combination of the most stable reference genes. Cq, mean quantification cycle; HG, high glucose; LPS, lipopolysaccharide; THP‐1, human acute leukemia‐derived monocyte

**Figure 10:**
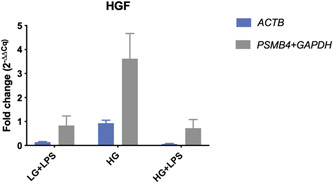
Expression fold changes (2^(−ΔΔCq)^) of *EEF1B2* between three experimental conditions and the low glucose (LG) group. Calculations were performed with a common housekeeping gene and a combination of the most stable reference genes. Cq, mean quantification cycle; HG, high glucose group; HGF, human gingival fibroblast; LPS, lipopolysaccharide

**Figure 11:**
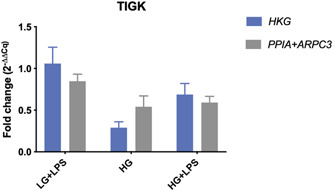
Expression fold changes (2^(−ΔΔCq)^) of *ACTB* between three experimental conditions and the low glucose (LG) group. Calculations were performed with a common housekeeping gene and a combination of the most stable reference genes. Cq, mean quantification cycle; HG, high glucose; LPS, lipopolysaccharide; TIGK, telomerase immortalized gingival keratinocyte

In the THP‐1 cell line, the relative fold change of *B2M* when normalized to *GAPDH* was 3.37 (±0.71) in the low‐glucose medium with LPS (LG + LPS) and 4.53 (±0.79) in the high‐glucose medium with LPS (HG + LPS). In contrast, when normalization was done with a proper set of reference genes (*ACTB* and *RPL10A)*, the fold changes decreased to 2.30 (±0.34) in the LPS‐supplemented low‐glucose medium and 2.20 (±0.28) in the respective high‐glucose medium (Figure 9 and Table 2). No substantial differences were witnessed in the high‐glucose conditions without LPS (HG). The HGF cell line showed fold changes below 1 (Table 2), indicating a reduction in gene expression respective to the low‐glucose condition (LG). While the expression of *EEF1B2* was seemingly slightly reduced by a 0.93 (±0.12) fold change between the LG and HG treatment when normalized to *ACTB*, the fold change increased to 3.62 (±1.05) when the proposed reference gene index (*PSMB4* and *GAPDH*) was applied. Moreover, the fold changes were altered from 0.14 to 0.83 in the LG + LPS group and from 0.06 to 0.72 in the HG + LPS group (Figure 10 and Table 2). On the other hand, the fold changes were rather indifferent in the TIGK cell line (Figure 11 and Table 2).

**Table 2:** Gene expression fold changes were calculated by efficiency‐corrected 2^−(ΔΔCq)^ method with a common housekeeping gene and the proposed reference gene index serving as the internal control

			HKG		RGI		
	Condition	Target	Expression fold change (2^(−ΔΔCq)^)	SEM	Expression fold change (2^(−ΔΔCq)^)	SEM	Δ2^(−ΔΔCq)^ (RGI‐HKG)
TIGK	LG + LPS	*ACTB*	1.058	0.479	0.848	0.207	−0.21
	HG		0.290	0.326	0.541	0.318	+0.25
	HG + LPS		0.686	0.170	0.592	0.181	−0.09
THP‐1	LG + LPS	*B2M*	3.374	0.713	2.300	0.338	−1.074
	HG		1.071	0.217	1.029	0.119	−0.042
	HG + LPS		4.534	0.789	2.205	0.283	−2329
HGF	LG + LPS	*EEF1B2*	0.144	0.019	0.831	0.4	+0.687
	HG		0.932	0.122	3.618	1.054	+2.686
	HG + LPS		0.06	0.021	0.721	0.362	+0.661

*Note*: The LG served as the control group, while the other experimental groups served as test groups.

Abbreviations: Cq, mean quantification cycle; HGF, human gingival fibroblasts; HKG, housekeeping gene; LG, low glucose group; THP‐1, human acute leukemia‐derived monocytes; TIGK, telomerase immortalized gingival keratinocytes; RGI, reference gene index; SEM, standard error of the mean.

## 4 DISCUSSION

In this study, we were able to demonstrate that commonly applied HKG may be liable to significant changes in expression, even by marginal variations of the experimental conditions. Also, we propose a decisive approach to identify and validate adequate reference genes for RT‐qPCR experiments in periodontal research. That way identified genes *ARPC3, RPL10A*, and *PPIA* turned out to exhibit higher stability than HKG in two out of three investigated cell lines. Furthermore, the presented workflow emphasizes the need for evidence‐based application of normalization algorithms, in order to avoid quantification bias.

It is well established that adequate validation of reference genes is a necessary step in establishing a robust RT‐qPCR assay. Furthermore, it has become obligatory information for publication of respective research (Bustin et al., 2009; Kozera & Rapacz, 2013). Accordingly, uncritical use of common HKG, without proper evidence of usability should be avoided, as experimental conditions may have significant regulatory influence (Klenke et al., 2016). A variety of studies confirmed this, for example, Selvey et al. (2001), who reported a dose‐dependent inhibition of *ACTB* by Matrigel treatment, or Glare et al. (2002), who verified a significant variability of *GAPDH* and *ACTB* in asthmatic airway tissues. Moreover, not only experimental conditions have proven influential on putative reference gene expression but also significant expression disparities across different tissues are reported in the literature. For example, a study evaluating the most adequate reference genes for orthodontic or periodontal in vitro experiments using periodontal ligament fibroblasts identified *TBP* as most stably expressed (Kirschneck et al., 2017). In contrast, in a similar study on neutrophils, conducted by Zhang et al. (2005), *TBP* appeared to be the least stable reference gene. Our results are in line with these findings, as we identified *PPIA* to be the most adequate reference for keratinocyte cell line TIGK in this experimental setup, while the same gene exhibited unacceptable variation in gingival fibroblasts (Figures 5 and 7 and Supporting information Tables). Consequently, a large number of the so‐called reference‐gene manuscripts have been published in the last decade (Cook et al., 2009; Kheirelseid et al., 2010; Remans et al., 2008; Riedel et al., 2014), highlighting the most adequate reference genes for a respective experiment, tissue or cell line. However, considering the notion that the validation process must be reported for every experiment, the purpose of such studies decreases substantially—even though many of these articles may have proven beneficial for researchers designing a new assay (Bustin et al., 2010). Moreover, most of these manuscripts investigated arbitrary sets of candidate genes, led by the assumption that the most stable ones would suffice. However, the fact that normalization against a regulated gene may result in false‐negative results (Bas et al., 2004; Schmittgen & Zakrajsek, 2000; Tricarico et al., 2002) underlines the requirement of a standardized and reasoned preselection of putative reference genes. Hence, Hruz et al. (2011) initiated a new tool, RefGenes, which provides researchers with context‐specific reference genes drawn from available microarray datasets. Moreover, they presented evidence for the appropriateness of the algorithm‐derived genes in RT‐qPCR experiments. The findings of our study support the applicability of this tool while accentuating the issues associated with the use of unvalidated reference genes, thus rendering this approach a potent refinement for the development of high‐resolute RT‐qPCR assays.

In the present study, three different normalization algorithms were applied: Normfinder, geNorm, and Bestkeeper. Regarding the results for each approach separately, the ranking orders appear inconsistent with each other (Figures 5, 6, 7). This finding is in line with other studies (Jacob et al., 2013; Kirschneck et al., 2017; Nazet et al., 2019) and may be explained by different modes of calculation: While the geNorm software requires Cq values to be transformed into linear scale data, the Bestkeeper calculations originate from raw Cq values. Considering this, using at least three different algorithms for validation circumvents the bias deflecting from the different mathematical approaches (Kozera & Rapacz, 2013). However, this recommendation is an extension to the MIQE guideline, which calls for evidence of just one normalization algorithm (Bustin et al., 2009).

To our surprise, we discovered that *ACTB* was ranked among the most suitable reference genes for THP‐1 cells, suggesting that HKG may indeed be eligible in some cases, provided the validation process shows evidence for it. In further contrast to our main results, the genes proposed by RefGenes displayed substantially higher overall variation in the HGF‐hTert cell line. This may be explained by the fact that some microarray datasets for specific experimental conditions are not available yet in Genevestigator (Hruz et al., 2011). Consequently, we had to apply additional arrays from related tissues, which may have compromised the cell‐line specificity. Moreover, the least variable RefGenes‐derived target, *PSMB4*, exhibited almost no correlation to a suggested index with *GAPDH* and *SDHA*, irrespective of good stability values in terms of standard deviation.

The consecutive calculations revealed substantially deviating gene expression values in respect to the chosen internal reference, again highlighting the need for proper normalization (Figures 9, 10, 11). In the HGF cell line, the hypothetical gene expression increased almost four‐fold when a proper reference gene index was applied, suggesting that normalization against the HKG *ACTB* would have resulted in a false‐negative result. In addition to this, the THP‐1 cell line exhibited differences in target gene expression by almost two‐fold in the LG + LPS treatment group. However, the application of the proposed reference gene index decreased the expressional fold change by almost half (Figure 9), suggesting that unvalidated use of the HKG *GAPDH* would have led to false‐positive results under the given experimental setup. Moreover, even though the differences between fold changes were comparably small in the TIGK cell line, it should be noted that the hypothetical target genes used in this study are genes that are not highly regulated after all. Also, we observed higher fold change differences in the HGF cell line. This is related to the higher variability of the gene investigated (*EEF1B2*), suggesting that higher regulated target genes may be more prone to expression bias in respect to reference gene normalization. These findings are in line with Dheda et al. (2005) who were able to provide evidence for significantly differing expression data as a result of normalization to different unvalidated HKGs. As a matter of principle, it is to be expected that the quantification bias increases if a highly regulated target gene is investigated. Furthermore, the increased assay noise may prevent the detection of small expression changes, thus leading to false‐negative results.

In conclusion, we demonstrated that the use of unvalidated reference genes for RT‐qPCR studies may evoke extensive bias due to undetected expression variation. In TIGK and THP‐1 cell lines, common HKG exhibited unacceptable expressional changes to small variations of experimental conditions. While HKG may prove appropriate in some cases, RefGenes allows for adequate and condition‐specific preselection of reference genes, which leads to a more reasoned and evidence‐based selection process. Despite the mentioned limitations, we, therefore, suggest the present approach to identify and validate satisfactory internal reference genes, particularly in periodontal research.

## CONFLICT OF INTERESTS

The authors declare that there are no conflict of interests.

## AUTHOR CONTRIBUTIONS

Daniel Diehl contributed to conception, design, data acquisition and interpretation, performed all statistical analyses, drafted, and critically revised the manuscript. Hagen S. Bachmann contributed to conception, design and interpretation, and critically revised the manuscript. Anton Friedmann contributed to the conception, design, and critically revised the manuscript. All authors gave their final approval and agree to be accountable for all aspects of the work.

## Supporting information

Supporting information.

## Data Availability

The data that support the findings of this study are available from the corresponding author upon reasonable request.
