# Supplementary material for: Evidence‐based selection of reference genes for RT‐qPCR assays in periodontal research
Source: Clin Exp Dent Res. 2022 Feb 1;8(2):473–84. doi: 10.1002/cre2.525 (PMC9033546; doi:10.1002/cre2.525)
Supplement: Supplementary file 1 — Supporting information. [file CRE2-8-473-s001.docx]

**Supplementary material**

**Evidence-based selection of reference genes for RT-qPCR assays in periodontal research**

Daniel Diehl ^*,1, 2^, Anton Friedmann ^2^, Hagen S. Bachmann ^1^

^1^ Institute of Pharmacology and Toxicology

Center for Biomedical Education and Research (ZBAF)

Faculty of Health, Witten/Herdecke University

Stockumer Straße 10
 58453 Witten, Germany

Tel.: +49 (0)2302 / 926-302

^2^ Department of Periodontology, School of Dentistry

Faculty of Health, Witten/Herdecke University

Alfred-Herrhausen Str. 45

58455 Witten, Germany

Tel.: +49 (0)2302 / 926-608

E-Mail: [Daniel.diehl@uni-wh.de](mailto:Daniel.diehl@uni-wh.de)

^*^Corresponding author

1. **Selection of adequate Microarray datasets in Refgenes software**


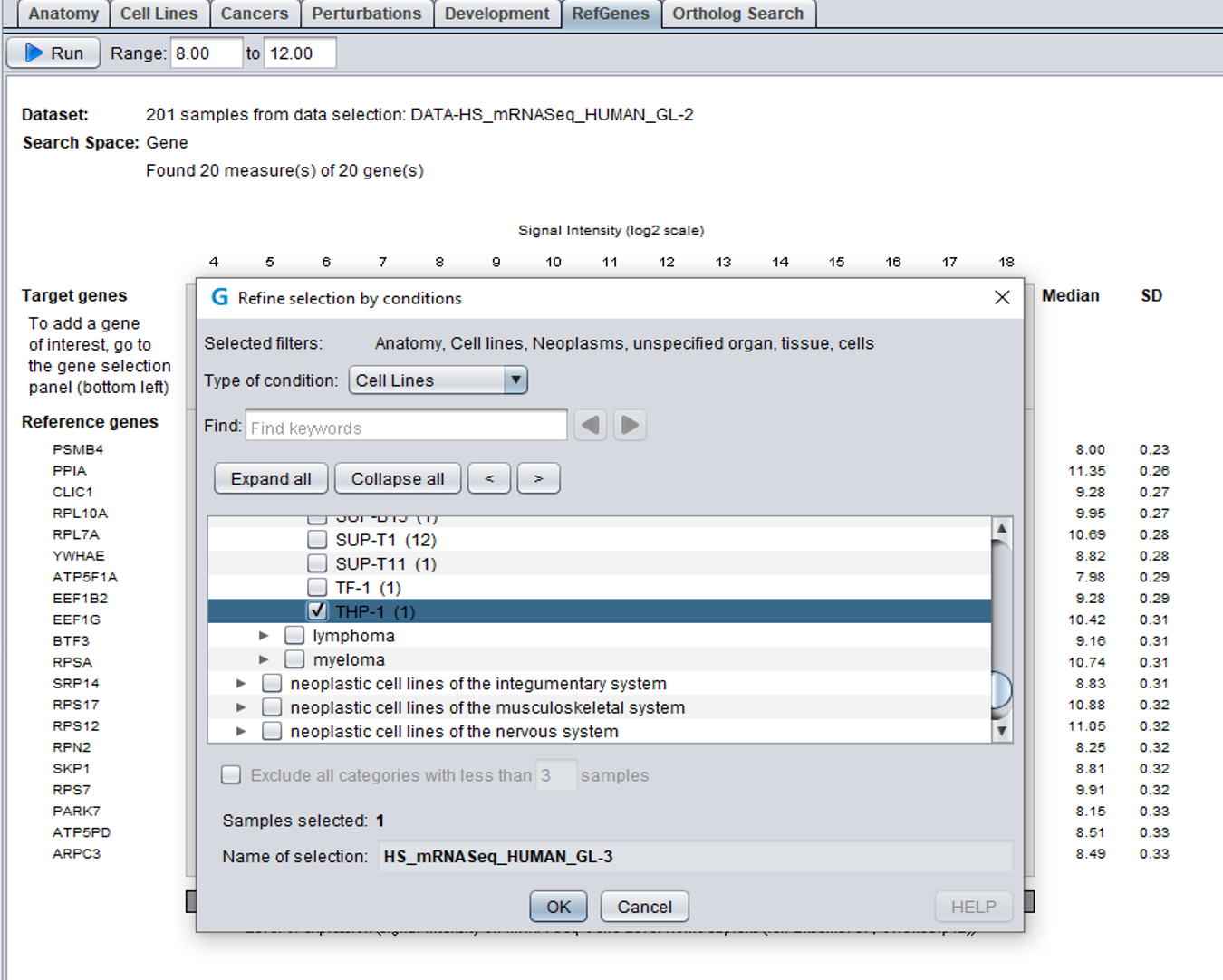


**Supplementary Figure 1a.** Selection of cell lines or conditions in RefGenes.


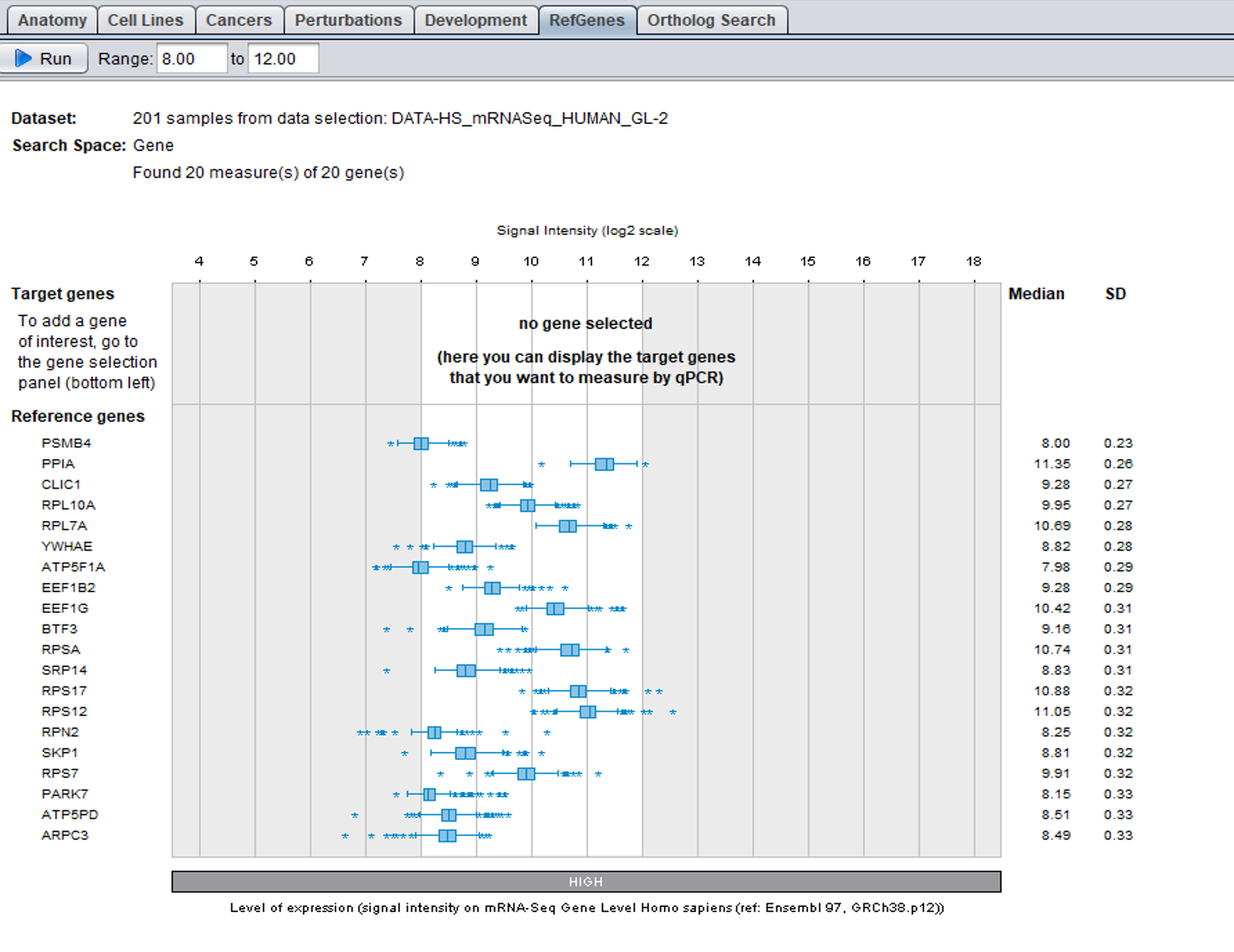


**Supplementary Figure 1b.** Target genes recommended by RefGenes software according to selected cell lines, tissues or perturbations.

1.
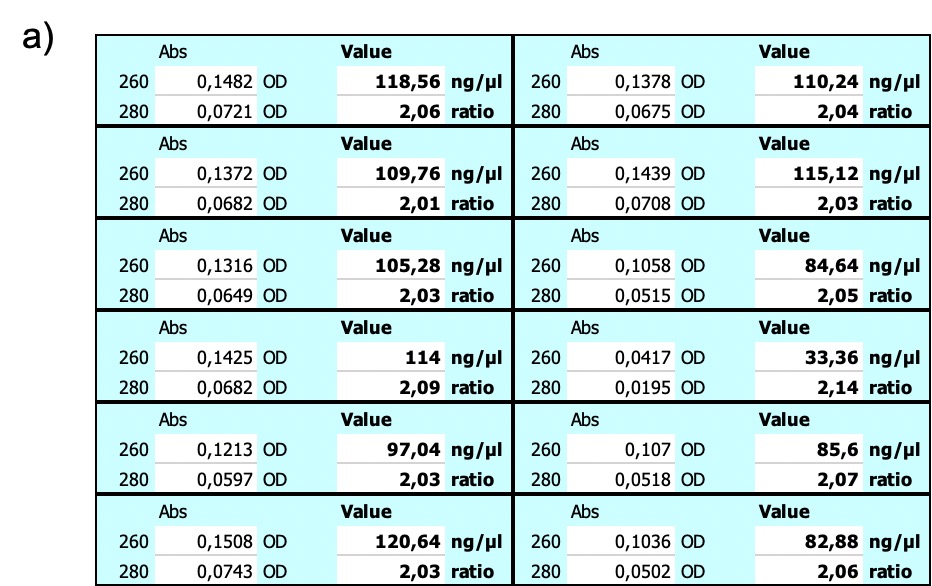
 **RNA quantification and quality assessment**

**Supplementary Table 1a.** HGF-hTert


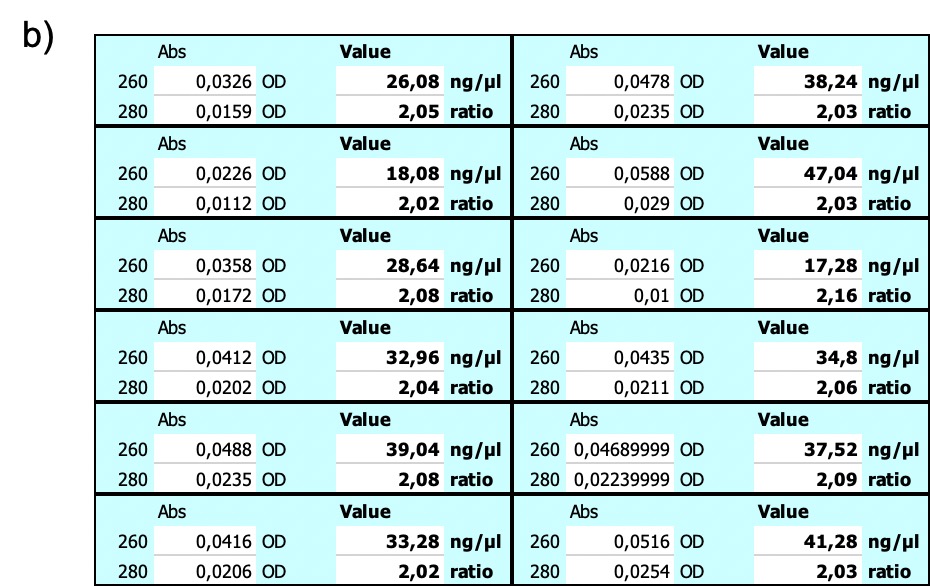


**Supplementary Table 1b**. THP-1


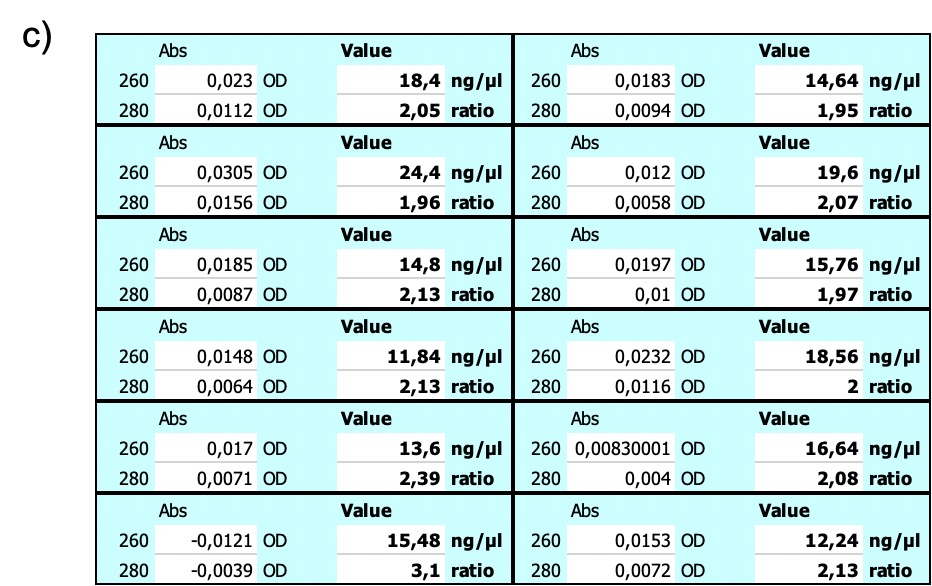


**Supplementary Table 1c.** TIGK.

1. **Full-length agarose gels**

**
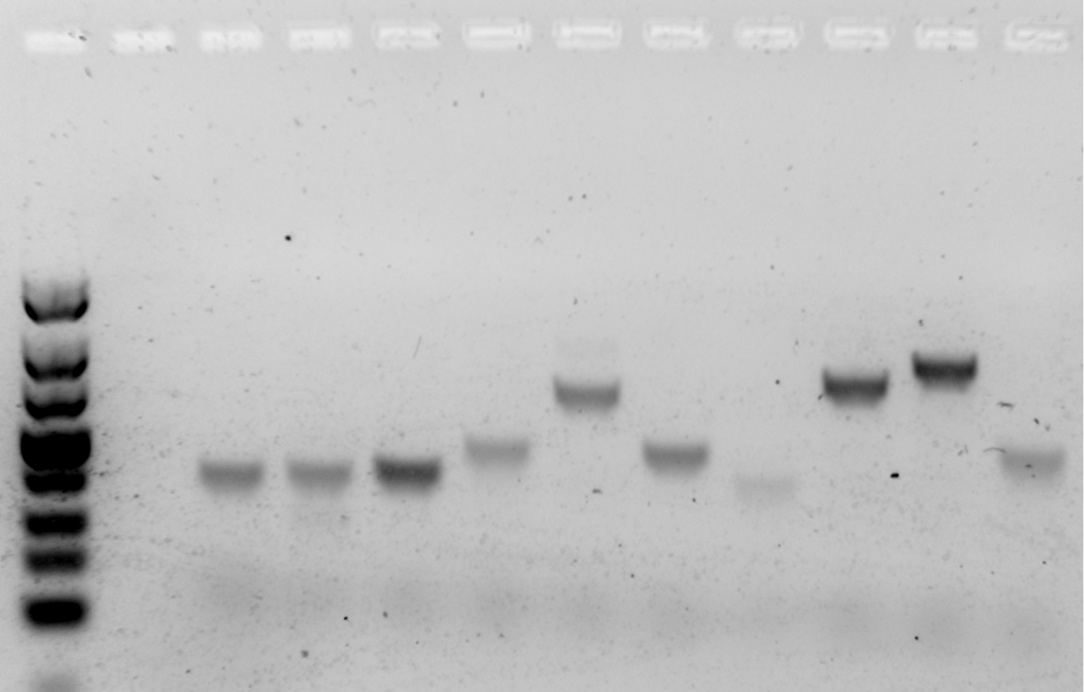
**

**Supplementary Figure 2a. hGF-hTert cell line**

**
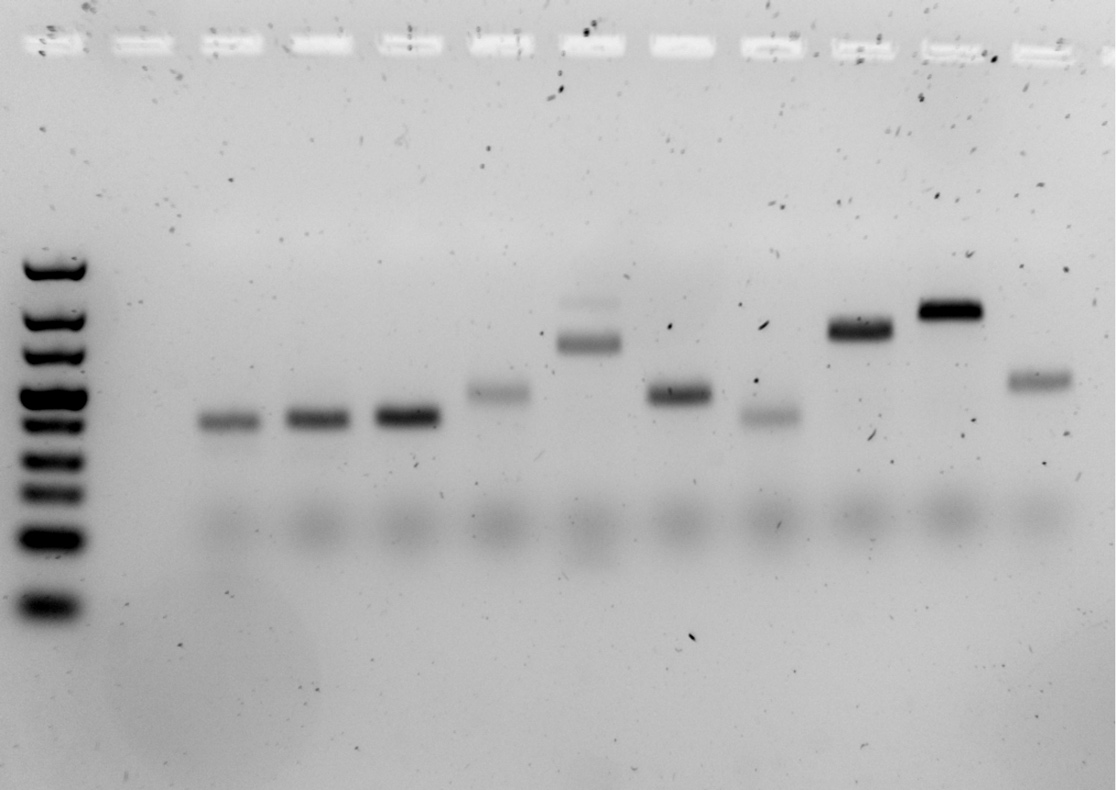
**

**Supplementary Figure 2b. THP-1 cell line**

**
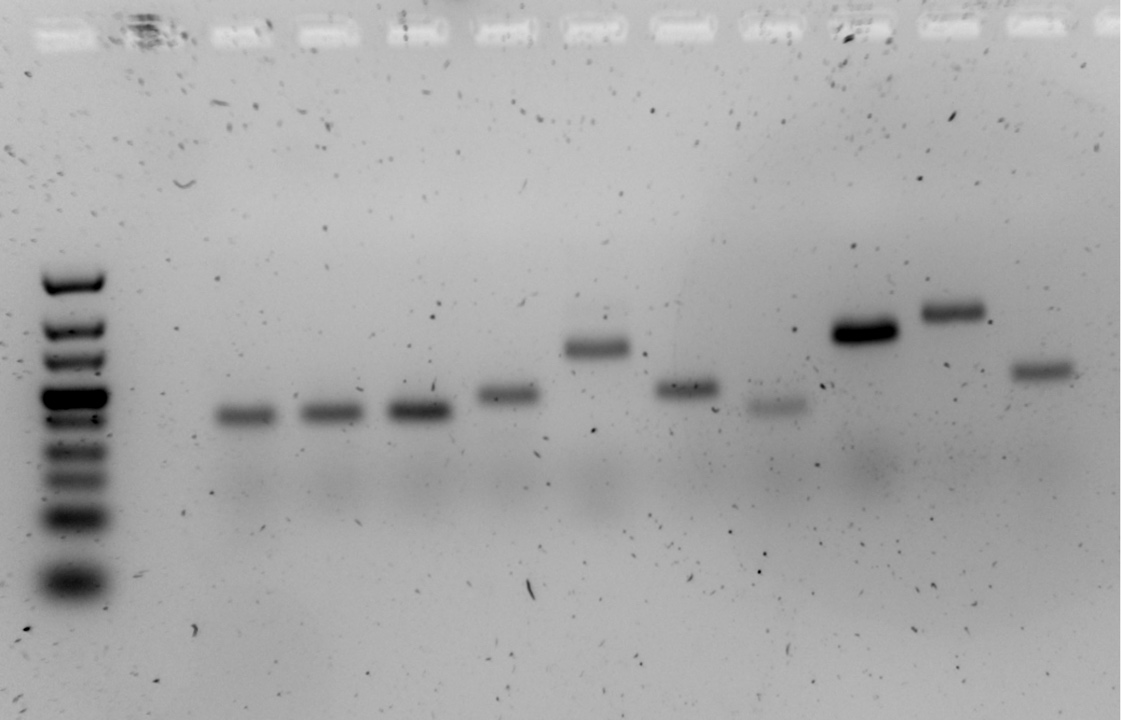
**

**Supplementary Figure 2c. TIGK cell line.**

1. **Primer efficiency and assay validation**


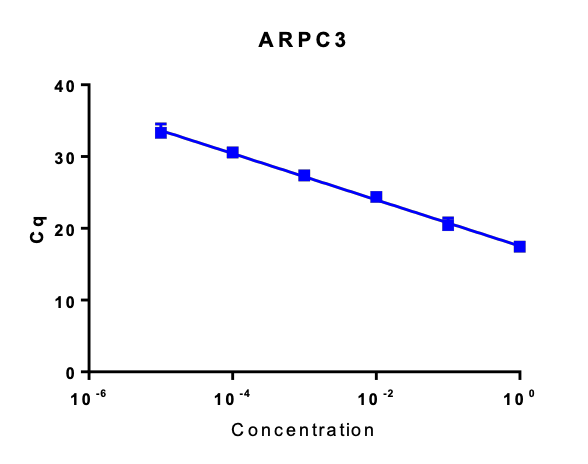


**Supplementary Figure 3.** Standard Curve for *ARPC3* derived from a 6x log_10_ serial dilution.

| Slope [±SD] | -3,224 ± 0,07828 |
| --- | --- |
| Amplification factor E | 2.04 |
| LDR (log.) | 10^0^-10^-5^ |
| LOD | 10^-6^ |
| r^2^ | 0,9976 |
| Annealing temperature T_a_ | 60.0°C |

**Supplementary Table 2.** Keystone values for Primer und Assay validation.


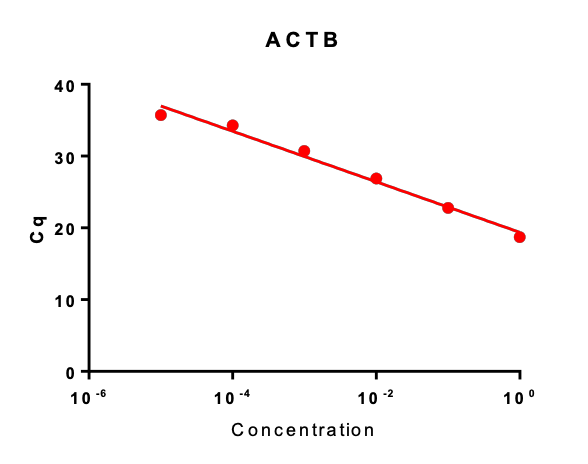


**Supplementary Figure 4.** Standard Curve for *ACTB* derived from a 6x log_10_ serial dilution.

| Slope [±SD] | -3,523 ± 0,223 |
| --- | --- |
| Amplification factor E | 1.92 |
| LDR (log.) | 10^0^-10^-5^ |
| LOD | 10^-6^ |
| r^2^ | 0,9842 |
| Annealing temperature T_a_ | 62.5°C |

**Supplementary Table 3.** Keystone values for Primer und Assay validation.


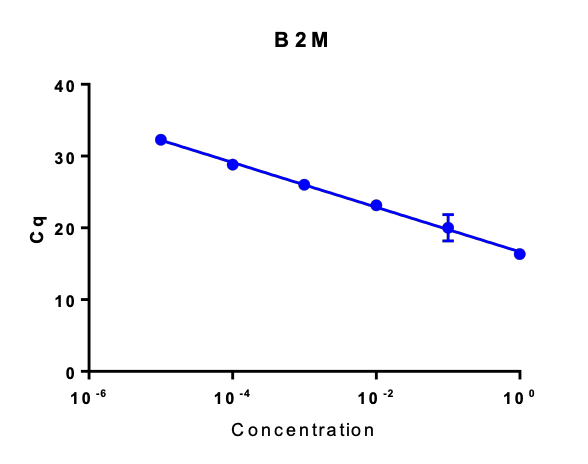


**Supplementary Figure 5.** Standard Curve for *B2M* derived from a 6x log_10_ serial dilution.

| Slope [±SD] | -3,113 ± 0,06654 |
| --- | --- |
| Amplification factor E | 2.10 |
| LDR (log.) | 10^0^-10^-5^ |
| LOD | 10^-6^ |
| r^2^ | 0,9982 |
| Annealing temperature T_a_ | 57.5°C |

**Supplementary Table 4.** Keystone values for Primer und Assay validation.


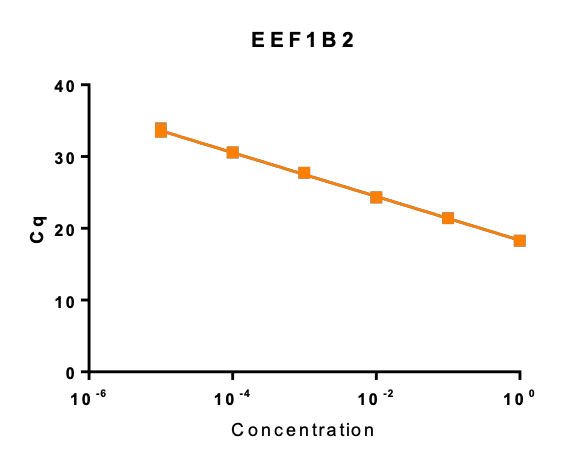


**Supplementary Figure 6.** Standard Curve for *EEF1B2* derived from a 6x log_10_ serial dilution.

| Slope [±SD] | -3,055 ± 0,03771 |
| --- | --- |
| Amplification factor E | 2.12 |
| LDR (log.) | 10^0^-10^-5^ |
| LOD | 10^-6^ |
| r^2^ | 0,9994 |
| Annealing temperature T_a_ | 56.0°C |

**Supplementary Table 5.** Keystone values for Primer und Assay validation.


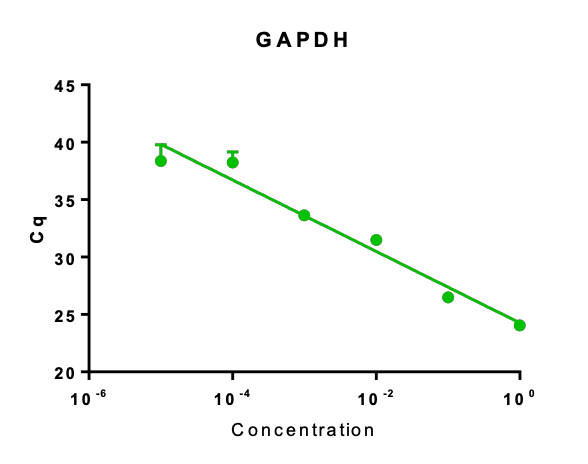


**Supplementary Figure 7.** Standard Curve for *GAPDH* derived from a 6x log_10_ serial dilution.

| Slope [±SD] | -3,112 ± 0,2988 |
| --- | --- |
| Amplification factor E | 2.10 |
| LDR (log.) | 10^0^-10^-5^ |
| LOD | 10^-6^ |
| r^2^ | 0,9967 |
| Annealing temperature T_a_ | 59.0°C |

**Supplementary Table 6.** Keystone values for Primer und Assay validation.


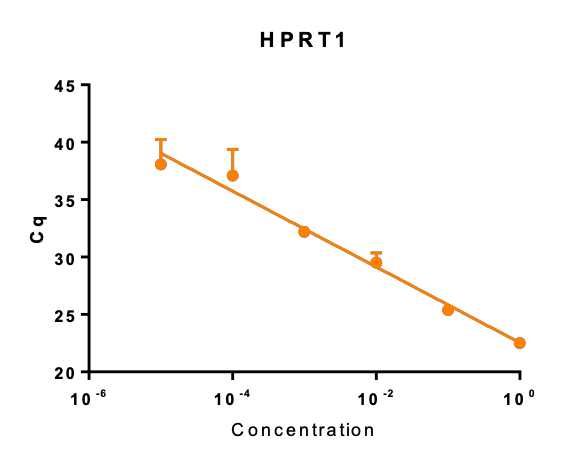


**Supplementary Figure 8.** Standard Curve for *HPRT1* derived from a 6x log_10_ serial dilution.

| Slope [±SD] | -3,303 ± 0,2136 |
| --- | --- |
| Amplification factor E | 2.00 |
| LDR (log.) | 10^0^-10^-5^ |
| LOD | 10^-6^ |
| r^2^ | 0,9835 |
| Annealing temperature T_a_ | 62.5°C |

**Supplementary Table 7.** Keystone values for Primer und Assay validation.


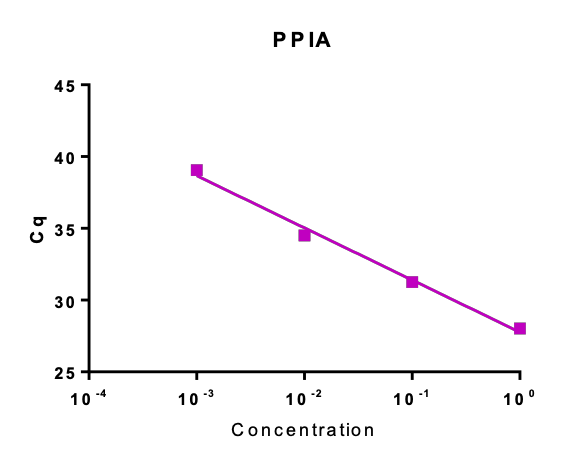


**Supplementary Figure 9.** Standard Curve for *PPIA* derived from a 6x log_10_ serial dilution.

| Slope [±SD] | -3,635 ± 0,2282 |
| --- | --- |
| Amplification factor E | 1.88 |
| LDR (log.) | 10^0^-10^-3^ |
| LOD | 10^-4^ |
| r^2^ | 0,9922 |
| Annealing temperature T_a_ | 62.5°C |

**Supplementary Table 8.** Keystone values for Primer und Assay validation.


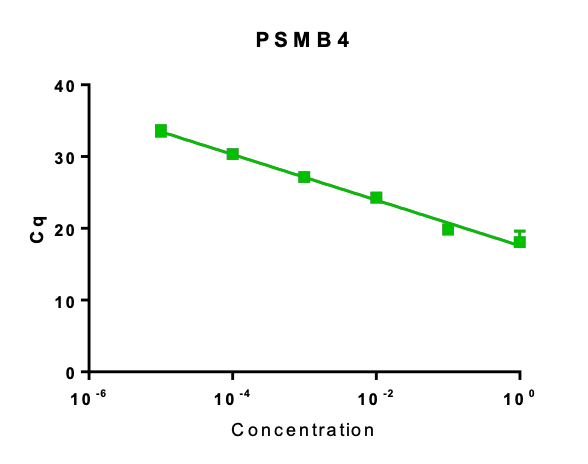


**Supplementary Figure 10.** Standard Curve for *PSMB4* derived from a 6x log_10_ serial dilution.

| Slope [±SD] | -3,635 ± 0,2282 |
| --- | --- |
| Amplification factor E | 1.88 |
| LDR (log.) | 10^0^-10^-3^ |
| LOD | 10^-4^ |
| r^2^ | 0,9922 |
| Annealing temperature T_a_ | 56.0°C |

**Supplementary Table 9.** Keystone values for Primer und Assay validation.


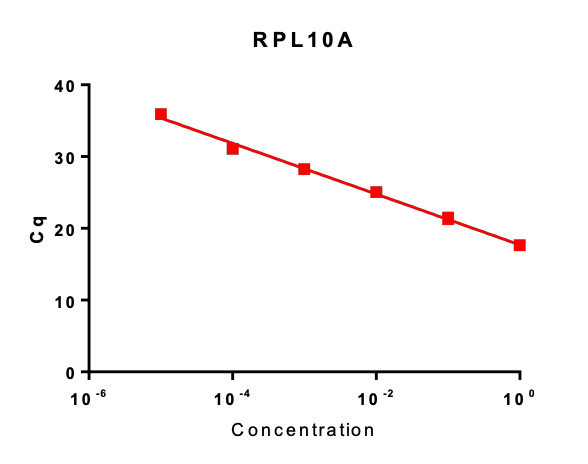


**Supplementary Figure 11.** Standard Curve for *RPL10A* derived from a 6x log_10_ serial dilution.

| Slope [±SD] | -3,538 ± 0,1182 |
| --- | --- |
| Amplification factor E | 1.92 |
| LDR (log.) | 10^0^-10^-3^ |
| LOD | 10^-4^ |
| r^2^ | 0,9956 |
| Annealing temperature T_a_ | 59.0°C |

**Supplementary Table 10.** Keystone values for Primer und Assay validation.


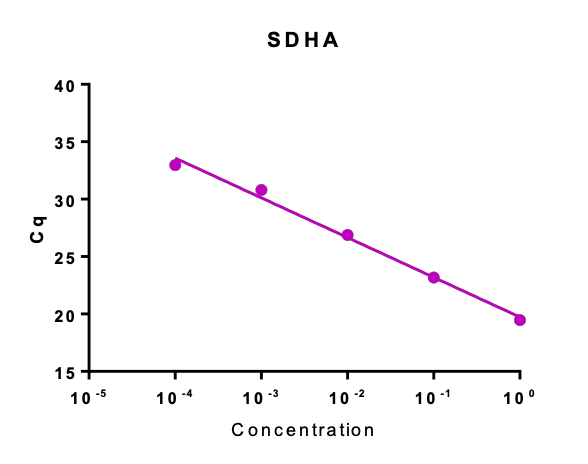


**Supplementary Figure 12.** Standard Curve for *SDHA* derived from a 6x log_10_ serial dilution.

| Slope [±SD] | -3,462 ± 0,1803 |
| --- | --- |
| Amplification factor E | 1.94 |
| LDR (log.) | 10^0^-10^-4^ |
| LOD | 10^-5^ |
| r^2^ | 0,9919 |
| Annealing temperature T_a_ | 62.5°C |

**Supplementary Table 11.** Keystone values for Primer und Assay validation.

1. **Evaluations of expression stability by means of different algorithms**

|  | **Bestkeeper** | | **Normfinder** | | **geNorm** | |
| --- | --- | --- | --- | --- | --- | --- |
|  | **Gene** | **SD** | **Gene** | **SD** | **Gene** | **M** |
| 1 | *RPL10A* | 0.30 | *PPIA* | 0.09 | *PPIA* | 0.085 |
| 2 | *PPIA* | 0.43 | *ARPC3* | 0.17 | *ARPC3* | 0.085 |
| 3 | *ARPC3* | 0.47 | *B2M* | 0.20 | *B2M* | 0.226 |
| 4 | *EEF1B2* | 0.54 | *HPRT1* | 0.35 | *HPRT1* | 0.287 |
| 5 | *B2M* | 0.56 | *RPL10A* | 0.38 | *PSMB4* | 0.316 |
| 6 | *GAPDH* | 0.57 | *SDHA* | 0.49 | *RPL10A* | 0.357 |
| 7 | *SDHA* | 0.66 | *PSMB4* | 0.50 | *GAPDH* | 0.419 |
| 8 | *ACTB* | 0.67 | *GAPDH* | 0.52 | *SDHA* | 0.477 |
| 9 | *HPRT1* | 0.71 | *EEF1B2* | 0.56 | *ACTB* | 0.517 |
| 10 | *PSMB4* | 0.72 | *ACTB* | 0.58 | *EEF1B2* | 0.547 |

**Supplementary Table 13.** Stability measurements of candidate reference genes in TIGK cells by means of three different algorithms. Bestkeeper and Normfinder express stability by standard deviation (SD) across experimental conditions, while geNorm calculates pairwise Cq variation, given as

M.

|  | **Bestkeeper** | | **Normfinder** | | **Genorm** | |
| --- | --- | --- | --- | --- | --- | --- |
|  | **Gene** | **SD** | **Gene** | **SD** | **Gene** | **M** |
| 1 | *ARPC3* | 0.20 | *RPL10A* | 0.031 | *RPL10A* | 0.061 |
| 2 | *HPRT1* | 0.24 | *ACTB* | 0.031 | *ACTB* | 0.061 |
| 3 | *GAPDH* | 0.28 | *PSMB4* | 0.034 | *PSMB4* | 0.084 |
| 4 | *PSMB4* | 0.30 | *SDHA* | 0.207 | *SDHA* | 0.133 |
| 5 | *ACTB* | 0.36 | *ARPC3* | 0.272 | *ARPC3* | 0.200 |
| 6 | *RPL10A* | 0.39 | *HPRT1* | 0.291 | *HPRT1* | 0.238 |
| 7 | *PPIA* | 0.50 | *PPIA* | 0.384 | *PPIA* | 0.285 |
| 8 | *SDHA* | 0.50 | *GAPDH* | 0.496 | *GAPDH* | 0.327 |
| 9 | *B2M* | 0.78 | *EEF1B2* | 0.717 | *EEF1B2* | 0.413 |
| 10 | *EEF1B2* | 1.41 | *B2M* | 0.744 | *B2M* | 0.482 |

**Supplementary Table 14.** Stability measurements of candidate reference genes in THP-1 cells by means of three different algorithms. Bestkeeper and Normfinder express stability by standard deviation (SD) across experimental conditions, while geNorm calculates pairwise Cq variation, given as M.

|  | **Bestkeeper** | | **Normfinder** | | **Genorm** | |
| --- | --- | --- | --- | --- | --- | --- |
|  | **Gene** | **SD** | **Gene** | **SD** | **Gene** | **M** |
| 1 | *GAPDH* | 0.34 | *B2M* | 0.56 | *PSMB4* | 0.140 |
| 2 | *PSMB4* | 0.35 | *GAPDH* | 0.79 | *ARPC3* | 0.140 |
| 3 | *SDHA* | 0.51 | *SDHA* | 1,00 | *RPL10A* | 0.246 |
| 4 | *HPRT1* | 0.55 | *HPRT1* | 1.20 | *PPIA* | 0.318 |
| 5 | *B2M* | 0.74 | *ARPC3* | 1.39 | *B2M* | 0.763 |
| 6 | *ACTB* | 0.78 | *ACTB* | 1.40 | *GAPDH* | 1.163 |
| 7 | *EEF1B2* | 0.88 | *PSMB4* | 1.43 | *SDHA* | 1.373 |
| 8 | *ARPC3* | 1.17 | *PPIA* | 1.44 | *HPRT1* | 1.480 |
| 9 | *PPIA* | 1.28 | *RPL10A* | 1.71 | *ACTB* | 1.536 |
| 10 | *RPL10A* | 1.34 | *EEF1B2* | 2.26 | *EEF1B2* | 1.708 |

**Supplementary Table 15.** Stability measurements of candidate reference genes in HGF cells by means of three different algorithms. Bestkeeper and Normfinder express stability by standard deviation (SD) across experimental conditions, while geNorm calculates pairwise Cq variation, given as M.
